# An autoinducible trp‐T7 expression system for production of proteins and biochemicals in *Escherichia coli*

**DOI:** 10.1002/bit.27297

**Published:** 2020-02-18

**Authors:** Jenny Landberg, Hemanshu Mundhada, Alex Toftgaard Nielsen

**Affiliations:** ^1^ The Novo Nordisk Foundation Center for Biosustainability Technical University of Denmark Kongens Lyngby Denmark; ^2^ CysBio ApS Hørsholm Denmark

**Keywords:** *Escherichia coli*, inducible promoter, l‐serine, tryptophan

## Abstract

Inducible expression systems can be applied to control the expression of proteins or biochemical pathways in cell factories. However, several of the established systems require the addition of expensive inducers, making them unfeasible for large‐scale production. Here, we establish a genome integrated trp‐T7 expression system where tryptophan can be used to control the induction of a gene or a metabolic pathway. We show that the initiation of gene expression from low‐ and high‐copy vectors can be tuned by varying the initial concentration of tryptophan or yeast extract, and that expression is tightly regulated and homogenous when compared with the commonly used lac‐T7 system. Finally, we apply the trp‐T7 expression system for the production of l‐serine, where we reach titers of 26 g/L in fed‐batch fermentation.

## Graphical abstract

Inducible expression of biochemicals and proteins is widely used. Here, a tryptophan‐controlled, tightly regulated and tuneable T7‐based induction system is described and shown to be compatible with fed‐batch fermentation.

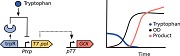

## 1 INTRODUCTION

Cell factories are promising options for the sustainable production of biobased compounds such as proteins and biochemicals (Nielsen & Keasling, 2016). While some products have been commercialized, it is generally difficult to produce those compounds at a price that is competitive with petroleum‐derived chemicals (Gustavsson & Lee, 2016; Liu, Zhu, Tan, Zhang, & Ma, 2016). The complex (heterologous) proteins and pathways that are used in cell factories oftentimes compete with native metabolic processes for cellular resources and pathway expression at an early growth phase can drain the cell for energy or result in redox imbalance (Blazeck & Alper, 2013; Brockman & Prather, 2015). Furthermore, if the end‐product is a toxic metabolite or protein, production at the beginning of the fermentation results in growth inhibition or poor growth (Saida, Uzan, Odaert, & Bontems, 2006). Expressing the product pathway from a promoter that is induced at a specific timepoint enables a two‐phase production process with biomass buildup followed by product accumulation. This approach can increase yield, titer, and productivity and improve process economics (Burg et al., 2016).

There are several inducible promoters and expression systems available for use in *Escherichia coli* (Terpe, 2006). One of the most commonly used systems is the lac‐T7 (DE3) system (Studier & Moffatt, 1986). It consists of a T7 RNA polymerase (T7 polymerase) controlled by the *lac*UV5 promoter and repressed by the lactose operon repressor (LacI). The protein or biochemical pathway is placed under control of the T7 promoter (*pT7*) and expression is induced by the addition of isopropyl β‐d‐1‐thiogalactopyranoside (IPTG) or by growth on lactose (Studier, 2005; Studier & Moffatt, 1986). Gene expression by T7 polymerase enables very high expression levels, which has led to extensive use of the system for protein production (Rosano & Ceccarelli, 2014). Proteins or biochemical pathways are also commonly expressed directly from the lac promoter (*Plac*; Gronenborn, 1976) or from the engineered lac and tryptophan fusion promoters Tac (de Boer, Comstock, & Vasser, 1983) and Trc (Brosius, Erfle, & Storella, 1985), which have lower basal‐ and higher expression levels compared with *Plac*. All these systems require the addition of IPTG for induction, which is expensive and unfeasible during the production of low‐ or medium‐value compounds (Keasling, 2012). Although IPTG‐based systems can also be induced by switching to lactose‐based growth (Studier, 2005), feeding lactose as a sole carbon source to a fed‐batch or continuous fermentation is not feasible due to its cost and relatively low solubility in water.

Inducible promoters can be tuned to improve their function and dynamic range. Common approaches to minimize basal level expression and tune the induced expression level include engineering/mutating the repressor, operator and upstream elements, or the −10 and −35 binding sites of the RNA polymerase as well as the spacing in between (Brosius et al., 1985; Chen et al., 2018; Ellefson, Ledbetter, & Ellington, 2018; Guan, Liu, Li, & Zhang, 2013). It has also been shown that novel, synthetic, ligand‐responsive promoter sequences can be used to improve expression levels up to 100‐fold compared with native sequences (Liu et al., 2019). If the promoter function is unknown, the characterization of important promoter elements can be done by establishing mutant promoter libraries and screening those in a high‐throughput manner (Rohlhill, Sandoval, & Papoutsakis, 2017). Using information gained in a promoter library screen, the repressor binding site of the formaldehyde‐inducible promoter could be identified and the information could be used to engineer mutated promoter variants with lower basal‐ and higher induced level expression and a significantly improved dynamic range (Rohlhill et al., 2017). Thermodynamic models are also helpful in the design of inducible promoters, especially to elucidate and predict the interaction between RNA polymerase and DNA (Brewster, Jones, & Phillips, 2012; Chen et al., 2018). In a recent study, Chen et al. (2018) developed a method that can be used to engineer the dynamic range of ligand‐inducible promoters. Using a thermodynamic model, they established a promoter library with different −10 and −35 sites and applied the optimal site combinations to build promoters regulated by different combinations of transcription factors, resulting in predictable expression with a variable dynamic range.

Several attempts have been made to find affordable and easy‐to‐apply (auto)inducible expression systems with low levels of basal expression and high, tunable levels of gene expression. Such systems do for example include those based on sensing of temperature, cell signaling, or depletion/addition of a compound that is naturally occurring in the cultivation medium. For temperature‐based induction, the *p*_*L*_ and *p*_*R*_ phage promoters can be used to induce gene expression, usually by shifting temperature from below 37 to up to 42°C (Elvin et al., 1990; Kincade & deHaseth, 1991). The system has been coupled to the T7 polymerase for high‐level protein production of protein (Chao, Law, Chen, & Hung, 2002). Although an inexpensive method of control, increased cultivation temperatures can induce cellular stress responses and alter protein folding (Valdez‐Cruz, Caspeta, Pérez, Ramírez, & Trujillo‐Roldán, 2010). In addition, exact and homogenous temperature control can be difficult to achieve in large‐scale vessels (Gvazdaitjs et al., 1994). For cell signaling‐based induction, the native bacterial quorum‐sensing system has been applied to establish autoinducible production by coupling gene expression to the concentration of secreted quorum signaling molecules (Gupta, Reizman, Reisch, & Prather, 2017; Kim et al., 2017). This type of system has also been shown to work when coupled to the T7 polymerase for further amplification of protein output (Zargar, Quan, & Bentley, 2016). While the expression is not always tightly regulated and the system might require tuning for each desired compound, further engineering could provide a system suitable for large‐scale fermentation.

The tryptophan promoter (*Ptrp*) is induced as tryptophan is depleted from the medium, or by addition of β‐indoleacrylic acid. It has been extensively applied for protein and polypeptide production (Bass & Yansura, 2000) and there are several expression vector systems based on the promoter (Chevalet, Robert, Gueneau, Bonnefoy, & Nguyen, 2000; Rosano & Ceccarelli, 2014). Generally, the native or leaderless (without the trp leader sequence *trpL*) promoter and the tryptophan repressor (*trpR*) have been inserted into an expression vector for direct control of a gene of interest (GOI). The promoter is tightly regulated, but does not provide as high expression levels as lac‐T7 and plasmid instability is prevalent (Somerville, 1988).

In this study, we engineered a genome integrated autoinducible expression system by combining the tightly regulated tryptophan promoter with the high‐expressing T7 polymerase. We showed that induction of gene expression could be tuned by the initial tryptophan concentration for low‐ and high‐copy vectors, and that expression strength was dependent on the use of the native or leaderless promoter constructs. By cloning the toxin MazF under control of our promoter constructs, we verified that the promoters are tightly regulated and suitable even for toxic protein production. Using flow cytometry, we further demonstrated that population homogeneity was improved compared with the commonly used lac‐T7 system. Finally, we showed that the trp‐T7 expression system can be used for biochemical production by applying it for batch and fed‐batch fermentation of l‐serine.

## 2 MATERIALS AND METHODS

### 2.1 Strains, media, and materials

For cultivation and screening during cloning, 2xYT (16 g/L Bacto tryptone, 10 g/L yeast extract (YE), 5 g/L NaCl) medium and lysogeny broth (LB; 10 g/L tryptone, 5 g/L YE, 10 g/L NaCl) agar plates with appropriate antibiotics were used. Ampicillin, kanamycin, chloramphenicol, and spectinomycin was used with working concentrations of 100, 50, 50, and 50 μg/mL, respectively. For cloning and screening of plasmids carrying a tryptophan repressible gene, 0.5 mM tryptophan was added to agar plates and growth medium to repress gene expression. Growth and production experiments were performed in M9 minimal medium with 0.1 mM CaCl_2_, 2.0 mM MgSO_4_, 1X trace element solution and 1X M9 salts supplemented with glucose, appropriate antibiotics and other compounds as described below. The stock solution of trace elements was 1,000X and consisted of 15 g/L EDTA(Na_2_)·2H_2_O, 4.5 g/L ZnSO_4_·7H_2_O, 0.7 g/L MnCl_2_·4H_2_O, 0.3 g/L CoCl_2_·6H_2_O, 0.2 g/L CuSO_4_·2H_2_O, 0.4 g/L NaMoO_4_·2H_2_O, 4.5 g/L CaCl_2_·2H_2_O, 3 g/L FeSO_4_·H_2_O, 1 g/L H_3_BO_3_, and 0.1 g/L KI dissolved in double‐distilled water and sterile filtered. The stock solution of M9 salts was 10X and consisted of 6.8 g/L Na_2_HPO_4_ anhydrous, 3 g/L KH_2_PO_4_, 5 g/L NaCl, and 1 g/L NH_4_Cl dissolved in double‐distilled water and autoclaved. The strains and plasmids that were used are listed in Table 1. Chemicals were purchased from Sigma‐Aldrich (Taufkirchen, Germany). Restriction enzymes and polymerase chain reaction (PCR) polymerases were purchased from Thermo Fisher Scientific (Waltham, MA). USER enzyme was purchased from BioNordika (Herlev, Denmark).

**Table 1:** Strains and plasmids used in the study

Strain	Description	Reference/source
*E. coli* NEB 5‐alpha	*fhuA2* Δ*(argF‐lacZ)U169 phoA glnV44 Φ80* Δ*(lacZ)M15 gyrA96 recA1 relA1 endA1 thi‐1 hsdR17*; cloning strain	New England Biolabs
*E. coli* K12 MG1655	F‐ lambda‐ *ilvG‐ rfb‐50 rph‐1*	Lab collection
*E. coli* MG1655 (DE3)	**lac‐T7**: MG1655 λ *(DE3)*	Mundhada, Schneider, Christensen, and Nielsen (2016)
ALE5	MG1655 Δ*sdaA*Δ*sdaB*Δ*tdcG*Δ*glyA*; serine tolerant strain	Mundhada et al. (2017)
ALE5	MG1655 Δ*sdaA*Δ*sdaB*Δ*tdcG*Δ*glyA* λ*(DE3)*; serine tolerant strain	Mundhada et al. (2017)
JL13	**trp‐T7**: MG1655 attB‐186(O): P_trp_; tryptophan repressor and promoter controlling T7 polymerase expression	This study
JL44	trp*‐T7: MG1655 attB‐186(O): P_trp*_; tryptophan repressor and truncated promoter controlling T7 polymerase expression	This study
JL45	trp‐T7 with pSEVA27‐mCherry	This study
JL46	trp*‐T7 with pSEVA27‐mCherry	This study
JL84	**trp‐T7‐ser**: ALE5 attB‐186(O): P_trp_; tryptophan repressor and promoter controlling T7 polymerase expression	This study
JL141	trp‐T7 with pCDF‐mCherry	This study
JL142	trp*‐T7 with pCDF‐mCherry	This study
JL151	lac‐T7 with pSEVA‐mCherry	This study
JL153	lac‐T7 with pCDF‐mCherry	This study
JL154	trp‐T7‐ser with pSEVA27‐serABC	This study
JL155	trp*‐T7‐ser with pSEVA27‐serABC	This study
JL160	trp‐T7 with pSEVA27	This study
JL161	trp‐T7 with pSEVA27‐mazF	This study
JL162	trp‐T7 with pCDF	This study
JL163	trp‐T7 with pCDF‐mazF	This study
JL164	trp*‐T7 with pSEVA27	This study
JL165	trp*‐T7 with pSEVA27‐mazF	This study
JL166	trp*‐T7 with pCDF	This study
JL167	trp*‐T7 with pCDF‐mazF	This study

Plasmid	Description	Reference/source
pOSIP KO	Cloning‐integration plasmid, integrates at phage 186 site; Km^R^	St‐Pierre et al. (2013)
pOSIP KO‐lacI‐T7	Plasmid for integrating T7 polymerase under control of LacI; Km^R^	Mundhada et al. (2017)
pE‐FLP	Plasmid for expressing FLP recombinase to excise the integration plasmid backbone; Amp^R^	St‐Pierre et al. (2013)
pSEVA27‐sl3	**pSEVA27**: Low‐copy number plasmid pSC101 origin (∼5 copies per cell); MCS under T7 promoter; Km^R^	Kim, Cavaleiro, Rennig, and Nørholm (2016)
pCDF‐1b	**pCDF**: High‐copy number plasmid CloDF13 origin (∼20–40 copies per cell); MCS under T7 promoter; Sp^R^	Novagen
pJL3	pOSIP‐trp*‐T7: pOSIP KO harboring a P_trp*_ integration cassette controlling the T7 polymerase; Km^R^	This study
pJL5	**pSEVA‐mCherry**: SEVA27‐sl3 plasmid with mCherry expressed from the T7 promoter; Km^R^	This study
pJL9	**pOSIP‐trp‐T7**: pOSIP KO harboring a P_trp_ integration cassette controlling the T7 polymerase; Km^R^	This study
pJL42	pCDF‐1b plasmid with mCherry expressed from the T7 promoter; Sp^R^	This study
pJL83	**pCDF‐mCherry**: pCDF‐1b with *lacI* removed and mCherry expressed from the T7 promoter; Sp^R^	This study
pJL88	**pSEVA‐mazF**: pSEVA27‐sl3 plasmid with *mazF* expressed from the T7 promoter; Km^R^	This study
pJL89	**pCDF‐mazF**: pCDF‐1b plasmid with *lacI* removed and *mazF* expressed from the T7 promoter; Sp^R^	This study
pSEVA27‐sl‐*serA* ^mut^ *CB* TIR1	**pSEVA‐serACB**: Expression of the serine operon from the T7 promoter; Km^R^	Rennig et al. (2019)

*Note*: Names used to refer to strains and plasmids in the main text are written in bold.

Abbreviation: *E. coli*: *Escherichia coli*.

### 2.2 Plasmid and strain construction

Plasmids were purified using the Macherey Nagel plasmid purification kit (Dure, Germany). Cells were transformed using the TSS buffer method. Forty milliliters exponentially growing cells (OD ~0.4–0.8) were spun down and resuspended in 0.5–1 ml TSS buffer (100 g/L PEG 8000, 30 mM MgCl_2_, 20 ml dimethyl sulfoxide in LB). Cells were mixed with 100 ng plasmid and put on ice for 15 min. The plasmid‐cell mix was subjected to heat shock (42°C, 1 min) and put on ice for 2 min. Cells were incubated for 1 hr at 37°C before plating on LB agar with appropriate antibiotics. All plasmids were constructed by USER cloning (Cavaleiro, Kim, Seppälä, Nielsen, & Nørholm, 2015; Nour‐Eldin, Hansen, Nørholm, Jensen, & Halkier, 2006). Briefly, template DNA was amplified using Phusion U polymerase and primers containing a uracil complementary overhang. PCR products were purified and subjected to *Dpn*I digestion. The backbone and the template fragments were mixed with USER enzyme and buffer to a volume of 10 μl and incubated for 20 min at 37°C followed by 20 min at 25°C. The mix was diluted with 10 μl water and 10 μl of the diluted mix was used for transformation into competent cells. All primers used in the study were ordered from Integrated DNA Technologies (Leuven, Belgium) and are listed in Table S1. The tryptophan repressor, operator and promoter and the *mazF* gene were amplified from the *Escherichia coli* genome. Integration of trp‐T7 and trp*‐T7 was carried out according to the protocol by (St‐Pierre et al., 2013). Briefly, the cassettes were cloned on a pOSIP vector, followed by integration at the attB‐186(O) site. The antibiotic cassette was excised using an FLP recombinase.

### 2.3 Microtiter plate cultivations

Single transformants were inoculated in 96‐deep well plates in 500 μl M9 medium supplemented with 0.4% glucose, 0.02% YE, and 0.5 mM tryptophan. Cultures were grown overnight at 37°C, 250 rpm. Cells were washed and inoculated with a 1:100 inoculum ratio in microtiter plates with 150 μl M9 medium supplemented with 0.5% glucose and a specified amount of tryptophan or YE. The plates were sealed with oxygen‐permeable film (Breathe‐Easy sealing membrane, Sigma‐Aldrich) and incubated at medium shaking, 37°C in an ELx808 Absorbance Reader (BioTek, Winooski, VT) for OD_630_ measurements, or in a Synergy H1 Hybrid Multi‐Mode Reader (BioTek) for OD_630_ and fluorescence (excitation 587 nm, emission 617 nm) measurements.

### 2.4 Flow cytometry

Single transformants were inoculated in 2 ml M9 medium supplemented with 0.4% glucose, 0.02% YE, and 0.5 mM tryptophan. Cultures were grown overnight at 37°C, 250 rpm. Cells were washed and inoculated with an inoculum ratio of 1:100 in a 24‐deep well plate in 2.5 ml M9 medium supplemented with 0.4% glucose. The MG1655(DE3) strains were induced with 1 mM IPTG at OD_630_ approximately 0.3–0.4. Samples were diluted appropriately and analyzed in a MACSQuant® VYB flow cytometer (Miltenyi Biotec, Cologne, Germany). The expression of mCherry was detected using a yellow laser (561 nm) and the 615/20 nm Y2 filter. Forward (FSC) and side (SSC) scatter was detected with the yellow laser and a 561/10 nm filter. The results were analyzed with FlowJo (Becton, Dickinson and Company, Franklin Lakes, NJ).

### 2.5 Serine production in small batch fermentation

Preinoculums were prepared by inoculating single transformants in 2.5 ml M9 medium supplemented with 0.4% glucose, 0.02% YE, 0.5 mM tryptophan, and 2 mM glycine. Cultures were grown overnight at 37°C, 250 rpm. Cells were washed and inoculated to an OD of 0.05 in 50 ml M9 medium supplemented with 0.4% glucose, 2 mM glycine and 0.04 or 0.5 mM tryptophan.

### 2.6 Serine production in fed‐batch fermentation

Medium for fed‐batch fermentation was prepared as previously described (Mundhada et al., 2017), with an addition of 0.125% YE instead of 0.2% YE to the batch medium. A preculture was grown overnight at 37°C, 250 rpm, in 2xYT medium with 0.5 mM trp and 0.1% glucose. Cells were washed and the bioreactor was inoculated to an OD of 0.1. Oxygen saturation was maintained at 40% throughout the fermentation. Samples were taken regularly for analysis. A correlation of 0.374 between cell dry weight (CDW) and OD was used for CDW estimation and subsequent yield calculations (Mundhada et al., 2016).

### 2.7 Analytical methods

Samples from the small batch and fed‐batch fermentations were filtered and diluted appropriately. Glucose and byproducts were analyzed with high‐performance liquid chromatography as previously described (Kildegaard et al., 2014). Serine was analyzed with LC‐MS as previously described (Mundhada et al., 2016).

### 2.8 Data availability

All data is available from the authors upon request.

## 3 RESULTS AND DISCUSSION

### 3.1 Design and construction of a trp‐T7 expression system

**Figure 1:**
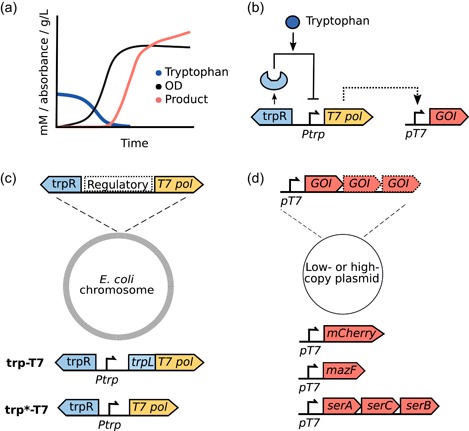
The layout and function of the trp‐T7 expression system. (a) Tryptophan‐based repression and induction for the production of a protein or biochemical. (b) Repression and induction of the trp‐T7 expression system is controlled by tryptophan concentration. When tryptophan is present in the growth medium it is bound by TrpR, which binds *trpO* and inhibits expression of T7 polymerase and the GOI. After tryptophan is consumed, the conformation of TrpR changes, leading to dissociation from *trpO*. T7 polymerase is produced and transcribes the plasmid‐based GOI from *pT7*. (c) The layout of the genome integrated cassettes trp‐T7 and trp*‐T7. (d) The plasmids with the different T7‐transcribed genes that were used to test trp‐T7‐based protein and biochemical production in this study. GOI, gene of interest; *trpO*, tryptophan operator; TrpR, tryptophan repressor [Color figure can be viewed at http://wileyonlinelibrary.com]

To establish a high‐expressing, inexpensive inducible system with low basal level expression, we wanted to combine the high level of gene expression achievable from the T7 polymerase with the tightly regulated trp operon. Using such a system, we hypothesized that the onset of gene expression could be tuned to a certain time point by regulating the amount of tryptophan added to the medium at the start of the cultivation (Figure 1a,b).

The trp operon consists of five structural genes encoding tryptophan synthase and is regulated by the TrpR. In growth medium with excess tryptophan, TrpR binds the *trpO*, blocking transcription of the operon from the *Ptrp*. The repression is further (~10‐fold) enhanced by attenuation, a mechanism where the *trpL* forms RNA stem‐loops that blocks transcription in the presence of tryptophan (Yanofsky, 1981). We constructed two integrative cassettes where the *E. coli* tryptophan regulation machinery was designed to control the T7 polymerase (Figure 1c). The first variant contained the tryptophan repressor and the native tryptophan leader sequence, promoter, and operator. This variant is hereafter referred to as trp‐T7. The second variant contained the tryptophan repressor, the tryptophan promoter and operator, but not the tryptophan leader sequence. This variant is hereafter referred to as trp*‐T7. The cassettes were cloned on a pOSIP integration vector (St‐Pierre et al., 2013) and integrated into the attB‐186(O) site in *E. coli* K12 MG1655, resulting in the two strains hereafter referred to as trp‐T7 and a trp*‐T7.

Low‐copy plasmid pSEVA27 (hereafter pSEVA) and high‐copy plasmid pCDF‐1b (hereafter pCDF) harboring *pT7* controlled by *lacO* were used as expression vectors to evaluate expression homogeneity and basal level expression, the timing of induction in different concentrations of tryptophan, and biochemical production from the expression system (Figure 1d). We decided to not replace the *lacO* site with a *trpO* site to facilitate cloning during future use of the system, as most T7‐based expression vectors have a *lacO* site present. We further hypothesized that *trpR*‐based repression of the genomic T7 polymerase would provide sufficient regulation of the system, as the trp operon is inherently under more strict regulation when compared with the lac operon. However, keeping *lacO* meant that we had to remove *lacI* from pCDF to enable target gene expression during growth on glucose.

### 3.2 Evaluation of expression profiles in different concentrations of tryptophan and YE

**Figure 2:**
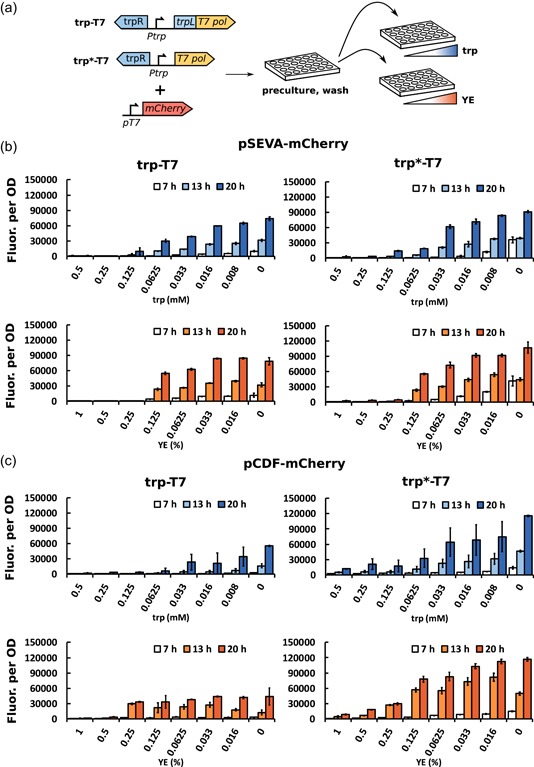
Experiment layout and fluorescence per OD after 7, 13, and 20 hr for strains harboring the trp‐T7 or trp*‐T7 expression system. The strains were grown with different starting concentrations of tryptophan (trp) or yeast extract (YE). (a) Experiment scheme. (b) Fluorescence per OD for strains trp‐T7 and trp*‐T7 with mCherry expressed from low‐copy plasmid pSEVA. (c) Fluorescence per OD for strains trp‐T7 and trp*‐T7 with mCherry expressed from high‐copy plasmid pCDF. The different time points are shown in bright (7 hr), medium (13 hr), or dark (20 hr) shades of blue or orange for strains grown with tryptophan or YE, respectively. The fluorescence per OD was calculated as the average of three biological replicates. The standard deviations are shown as error bars [Color figure can be viewed at http://wileyonlinelibrary.com]

For evaluation of the promoter system, strains with integrated trp‐T7 and trp*‐T7 (that contains or lacks the *trpL*, respectively), were transformed with a low‐ or high‐copy plasmid harboring mCherry under control of *pT7*, resulting in four different strains (Table 1 and Figure 1c,d). The induction profile of each strain was investigated using different starting concentrations of tryptophan and (tryptophan‐containing) YE with mCherry as a reporter gene (Figure 2a). Strains were grown in 0–0.5 mM tryptophan or 0–1% YE, and OD and fluorescence was monitored for 20 hr.

Overall, the initial concentration of tryptophan and YE was inversely correlated to the fluorescence per OD and the initiation of mCherry expression (Figures 2c,d and S1), showing that tryptophan and YE could be used to control the induction of the GOI in both versions of the trp‐T7 strains, and for both the high‐ and low‐copy vector. The fluorescence and fluorescence per OD at different time points were generally higher with decreasing concentrations of tryptophan or YE, since expression was induced earlier (Figure 2b,c). An exception was trp‐T7 with pCDF‐mCherry, where fluorescence per OD was similar for all YE concentrations between 0% and 0.25% (Figures 2c and S1). As expected, growth was dependent on the amount of YE added to the medium (Figure S1).

For strains carrying the low‐copy plasmid pSEVA‐mCherry (Figure 2b), high initial concentrations of tryptophan and YE (0.5, 0.25 mM, and 1%, 0.5%, and 0.25%, respectively) completely repressed mCherry expression in trp‐T7, while low levels of expression could be seen after approximately 10–13 hr in trp*‐T7. This is expected, as the *trpL*‐containing trp‐T7 version of the promoter should exhibit a higher level of repression than trp*‐T7. With most concentrations of tryptophan and YE, the fluorescence per OD was only marginally higher in trp*‐T7 compared to trp‐T7. However, for the strains carrying the high‐copy plasmid pCDF‐mCherry (Figure 2c), the fluorescence per OD was significantly higher in trp*‐T7, compared to trp‐T7, for both trp and YE cultures. Low but significant levels of mCherry were observed in trp*‐T7 even at high initial concentrations of tryptophan and YE, while, for trp‐T7, repression was similar to that seen in the low‐copy plasmid strains. Interestingly, the fluorescence per OD in strains harboring the trp‐T7 expression cassette was higher when mCherry was expressed from the low‐copy pSEVA plasmid than from high‐copy pCDF plasmid. This may be due to the higher metabolic burden resulting from maintaining multiple plasmid copies (Jones, Kim, & Keasling, 2000).

### 3.3 Comparing expression strength and homogeneity to lac‐T7

**Figure 3:**
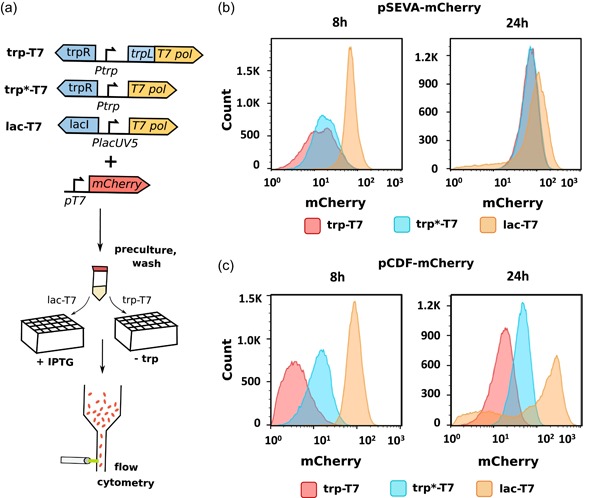
Histograms showing the fluorescence of strains trp‐T7, trp*‐T7, and lac‐T7 after 8 and 24 hr of growth. (a) Experiment scheme. (b) Fluorescence of strains with mCherry expressed from low‐copy vector pSEVA. (c) Fluorescence of strains with mCherry expressed from high‐copy vector pCDF. mCherry was induced by growth in tryptophan deplete medium (trp‐T7, trp*‐T7) or by addition of 1 mM IPTG (lac‐T7). Each histogram shown is a representative sample out of three biological replicates. IPTG, isopropyl β‐d‐1‐thiogalactopyranoside [Color figure can be viewed at http://wileyonlinelibrary.com]

Next, we sought to investigate the expression heterogeneity of both promoter constructs and compare it to the commonly used lac‐T7 expression system. A lac‐T7 strain (*E.coli* MG1655 (DE3); Mundhada et al., 2016) was transformed with pSEVA‐mCherry or pCDF‐mCherry. Overnight cultures were inoculated in minimal medium without tryptophan. The lac‐T7 cultures were induced with 1 mM IPTG at OD approximately 0.3–0.35. Expression levels were investigated with flow cytometry 8 and 24 hr after inoculation (Figure 3a).

For the pSEVA‐mCherry strains, fluorescence was slightly lower for trp‐T7 compared to trp*‐T7 after 8 hr (*p* = .027), while no significant difference was seen after 24 hr (*p* = .12) (Figures 3b and S2a). Fluorescence of the lac‐T7 strain was on average 3.9‐ and 2.7‐fold higher compared with the trp‐T7 and trp*T7 strain, respectively. The lac‐T7 strain also had a narrower distribution of fluorescence at the single‐cell level. The broad fluorescence profile seen in the trp strains could be the result of heterogeneous transport and degradation rates of tryptophan, resulting in differences in the trp operon induction response (Yanofsky, Horn, & Gollnick, 1991), and such population heterogeneity could be improved by for example engineering constitutive expression of tryptophan permeases. After 24 hr, the difference between the trp and lac strains had decreased, with the lac‐T7 strain having an average fluorescence that was 1.8‐ and 1.3‐fold higher compared with the trp‐T7 and trp*T7 strain, respectively. There was a subpopulation of low‐ and nonexpressing cells forming in the lac‐T7 strain, while expression in the trp strains was homogenous (Figures 3b and 2c).

For the pCDF‐mCherry strains, fluorescence was significantly higher for the trp*‐T7 compared with the trp‐T7 strain after 8 hr of growth (*p* = .009; Figures 3c and S2b). The lac‐T7 was expressing higher levels of mCherry than both trp strains, with a 17.9‐ and 5.5‐fold higher average fluorescence compared to trp‐T7 and trp*‐T7, respectively. Similar to the pSEVA‐mCherry cultures, the lac‐T7 strain had a narrower expression profile compared to both trp strains. After 24 hr, the difference between the trp and lac strains had decreased slightly, with lac‐T7 having 8.2‐ and 3.5‐fold higher average fluorescence levels compared with trp‐T7 and trp*‐T7, respectively (Figures 3c and S2b). However, mCherry expression in the lac‐T7 strain was highly heterogeneous, with a large population not expressing or expressing only low amounts of the protein (Figures 3c and S2d). The expression heterogeneity of the lac‐T7 system constitutes a significant problem when using the system in fed‐batch or continuous production processes. It results in decreased productivity, especially as the subpopulation that stops producing the compound of interest usually quickly outgrows the producing population (Rugbjerg, Sarup‐Lytzen, Nagy, & Sommer, 2018). Application of a less leaky version of the lac‐T7 system or application of a constitutive lac permease could potentially improve population homogeneity of the IPTG‐induced system, without affecting the expression levels negatively (Khlebnikov & Keasling, 2002).

Overall, the average fluorescence was significantly lower for trp‐T7 compared with the lac‐T7 strains (Figures 3b,c and S2a,b), which may partly be explained by the tight regulation of the trp expression system. In addition, due to the *lacO* site present on the expression plasmids, mCherry expression is also repressed by endogenously expressed *lacI*. Comparison of mCherry fluorescence levels for trp‐T7 and trp*‐T7 with or without IPTG induction showed that fluorescence per OD increased approximately 10% after IPTG addition for both pSEVA‐ and pCDF‐based expression vectors (data not shown). Thus, higher protein expression levels could potentially be achieved by deleting the endogenous *lacI* gene.

### 3.4 Evaluating the system for toxic protein production

Production of toxic proteins is challenging, as they can inhibit cell growth. An expression system with low basal level expression is preferred to avoid growth inhibition and decreased productivity when expressing such proteins. To investigate whether our constructs are suitable for toxic protein production, the *mazF* gene was cloned behind *pT7* on pSEVA and pCDF, resulting in plasmids pSEVA‐mazF and pCDF‐mazF. MazF is an ACA‐specific endoribonuclease that stalls cell growth by ribosome independent messenger RNA cleavage (Muñoz‐Gomez, Santos‐Sierra, Berzal‐Herranz, Lemmonier, & Mu, 2004; Zhang et al., 2003). The toxin has previously been used to demonstrate the suitability of inducible expression systems in *E. coli* (Guan et al., 2013). The *mazF* plasmids and respective control plasmids (no gene after *pT7*) were transformed to trp‐T7 and trp*‐T7, resulting in eight different strains.

**Figure 4:**
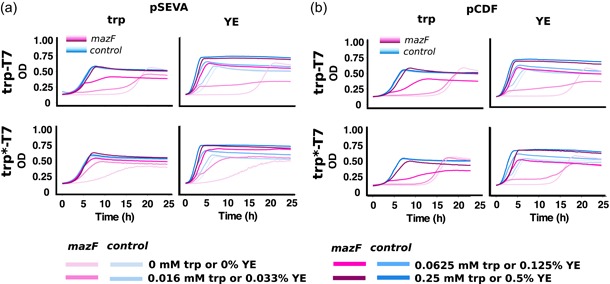
Growth curves of strains harboring the trp‐T7 expression systems and a mazF‐expressing plasmid (pink lines) or the corresponding control plasmid (blue lines). A darker shade of each color represents a higher concentration of trp or YE added to the medium at the beginning of the cultivation. (a) Growth curves of strains trp‐T7 and trp*‐T7 harboring *mazF* on pSEVA. (b) Growth curves of strains trp‐T7 and trp*‐T7 harboring *mazF* on pCDF. The growth curves were calculated as an average of three biological replicates. YE, yeast extract [Color figure can be viewed at http://wileyonlinelibrary.com]

The experiment was carried out similarly as shown in Figure 2a; overnight cultures were inoculated in microtiter plates with 0–0.25 mM trp or 0–0.5% YE and growth was monitored for 24 hr (Figure 4a,b). At the highest tryptophan and the two highest YE concentrations, no significant difference could be seen in growth rate and final OD between the control strains and the strains harboring the mazF plasmids, with the exception of trp*‐T7 harboring pCDF‐mazF where a slight decrease in growth rate could be seen also for the highest trp and YE concentrations (Figure 4b). At the lower concentrations, the MazF‐expressing strains did not grow, grew with a much lower growth rate or reached a lower OD compared with the control strain for all constructs. Overall, the results show that the trp‐T7 expression system is tight enough to be suitable for the expression of toxic proteins from high‐ and low‐copy vectors in *E. coli*.

Interestingly, growth was resumed in the toxin‐expressing strains toward the end of the microtiter plate cultivation. This phenomenon has been observed for the expression of other toxins in our lab and is possibly a result of single cells overcoming the toxic effect of MazF by mutating the toxin gene, the T7 polymerase or possibly by overexpressing the native antitoxin *mazE*. It is worth noting that growth was initiated at approximately the same timepoint in all cultures where growth was completely inhibited by toxin expression, indicating some common underlying mechanism.

### 3.5 Applying trp‐T7 for production of l‐serine

l‐serine is currently used in the pharmaceutical and cosmetics industry but has potential for extended industrial use as a building block for numerous chemicals (Werpy & Petersen, 2004). In previous studies, the l‐serine pathway has been expressed from *pT7* by an integrated *lac*UV5‐T7 polymerase induced by the addition of IPTG (Mundhada et al., 2016, 2017). To test l‐serine production from our tryptophan repressible expression system, the trp‐T7 cassette was integrated into an l‐serine‐tolerant strain of *E. coli* (Mundhada et al., 2017). We chose to only apply one of the cassettes, as mCherry production levels of trp‐T7 and trp*‐T7 were similar according to flow cytometry (Figure 3b). The strain was transformed with pSEVA27‐sl‐*serA* ^mut^ *CB* TIR1 (hereafter pSEVA‐serACB) harboring an expression‐optimized serine operon under control of *pT7* (Rennig et al., 2019), resulting in strain trp‐T7‐ser.

**Figure 5:**
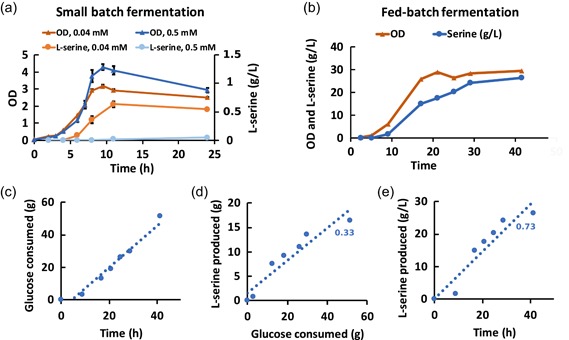
Serine production in trp‐T7‐ser harboring the trp‐T7 expression system and plasmid pSEVA‐serACB. (a) Small batch fermentation. Growth of trp‐T7‐ser in 0.04 mM tryptophan (triangles, dark orange) and 0.5 mM tryptophan (triangles, blue). l‐serine production of trp‐T7‐ser in 0.04 mM tryptophan (circles, orange) and 0.5 mM tryptophan (circles, bright blue). OD and serine concentrations were calculated as the average of three biological replicates. The standard deviations are shown as error bars. (b) Growth (orange) and production (blue) curves for fed‐batch fermentation of trp‐T7‐ser. (c) Glucose consumed during the fed‐batch fermentation of trp‐T7‐ser. (d) l‐serine yield during the fed‐batch fermentation of trp‐T7‐ser. (e) The productivity of the fed‐batch fermentation of trp‐T7‐ser [Color figure can be viewed at http://wileyonlinelibrary.com]

Overnight cultures of trp‐T7‐ser were inoculated in shake flasks with either 0.04 or 0.5 mM tryptophan to test the inducible and repressible function of the system, respectively. OD and serine were measured continuously for 24 hr. At the end of cultivation, serine titers reached 0.55 g/L (Figure 5a), slightly lower than the 0.62 g/L achieved when using lac‐T7 (Rennig et al., 2019). A decrease in growth rate and final OD could be seen for the serine‐producing strain, which may be due to carbon being utilized for serine production instead of growth.

To investigate the scalability of the process, we further tested the strain using fed‐batch fermentation. Tryptophan was replaced by YE for initial repression of the serine pathway, as this is a more plausible alternative for large‐scale fermentation. After calculating the tryptophan concentration in the YE and taking the larger cultivation volume in the bioreactor into account, 0.125% YE was added to the medium before the start of cultivation. An overnight culture was inoculated in a 1 L bioreactor containing 250 ml medium supplemented with 0.125% YE. The glucose feed was started after 9 hr (at OD ~9) and growth and serine production were monitored for approximately 42 hr (Figure 5b). At the end of the fermentation, 26 g/L l‐serine had been produced, approximately half of the 48 g/L reached when using lac‐T7 (Rennig et al., 2019). The productivity was 0.73 g/Lh (Figure 5e), approximately 70% of that reached with the lac‐T7 system. However, the same yield of l‐serine per glucose (0.33 and 0.30 g/g for trp‐T7 and lac‐T7, respectively) was achieved; the lower titer and productivity may partly be explained by a decreased glucose uptake and growth rate in our trp‐T7 strain (Figure 5c,d). Increasing the concentration of YE to 0.2%, which was used in the lac‐T7 fermentation, might improve growth rate and glucose uptake but may also affect the timing of induction of the l‐serine pathway. Another contributing factor could also be the lower expression levels from trp‐T7 compared with lac‐T7 that was observed previously (Figure 3b).

## 4 CONCLUSIONS

Timing the induction of protein and product pathway expression can improve bioprocess performance and can serve as an important mode of control during fermentation. Low‐cost expression systems are of special interest for large‐scale fermentation and for the production of low‐or medium‐value compounds. In this study, we demonstrated that the *E. coli* tryptophan promoter can be used to control T7‐based expression of a GOI and that induction can be tuned by varying tryptophan or YE concentration in the start medium. Tryptophan depletion did not seem to impact growth in our experiments. However, for the production of other types of biochemicals or proteins, the growth rate and tryptophan uptake rate may be affected, which would impact the dynamics of the expression system. This could especially be the case if the expressed (heterologous) pathway enzymes or products have a high tryptophan content. In those cases, engineering approaches may have to be taken to tune the system and adapt to the new conditions.

Production levels were lower compared with using the lac‐T7 expression system for both protein and biochemical production. Further improvement of the system could be made by engineering constitutive tryptophan uptake or using a *lacI* knockout strain to improve induction homogeneity or engineering the dynamic range using a mutant promoter library approach (Rohlhill et al., 2017). Overall, the low‐cost and tight regulation of the trp‐T7 system makes it an attractive alternative for large‐scale production of biochemicals and proteins.

## CONFLICT OF INTERESTS

The authors declare that there are no conflict of interests.

## Supporting information

Supporting information
