# Supplementary material for: An autoinducible trp‐T7 expression system for production of proteins and biochemicals in Escherichia coli
Source: Biotechnol Bioeng. 2020 Feb 18;117(5):1513–24. doi: 10.1002/bit.27297 (PMC7186829; doi:10.1002/bit.27297)
Supplement: Supplementary file 1 — Supporting information [file BIT-117-1513-s001.docx]

# Supporting information

# An autoinducible trp-T7 expression system for production of proteins and biochemicals in *Escherichia coli*

**Running title:** A trp-T7 expression system for *Escherichia coli*

Jenny Landberg^1^, Hemanshu Mundhada^1,2^, Alex Toftgaard Nielsen^1^

^1^The Novo Nordisk Foundation Center for Biosustainability, Technical University of Denmark, 2800 Kongens Lyngby, Denmark.

^2^Present address: CysBio ApS, Agern Allé 1, 2970 Hørsholm, Denmark.

**Funding information**

Novo Nordisk Foundation grant number NNF16CC0020908 and NNF10CC1016517.

**Correspondence**

Hemanshu Mundhada

CysBio ApS

Agern Allé 1

2970 Hørsholm Denmark

Email: [hemu@cysbio.com](mailto:hemu@cysbio.com)

## Supplementary Table 1

Table S1. Primers used in this study.

| **Nr.** | **Primer sequence 5’- 3’** | **Description** |
| --- | --- | --- |
| 2197 | AGCTGAGGUCGCCTCAGC | Forward primer to amplify pSEVA27together with 2733 for mCherry and *mazF* insertion |
| 2733 | AAACTGGTCUCCTTCTTAAAGTTAAACAAAATTATTTCTAGAG | Reverse primer to amplify pSEVA27 together with 2197 for mCherry and *mazF* insertion |
| 11707 | AGATGCAUGGCGCCTAACC | Forward primer to amplify pOSIP-trp-T7 together with jl26 to remove *trpL* |
| 11708 | AGAGGAUCCCCGGGTAC | Reverse primer to amplify pOSIP-KO-T7 together with 11834 for *trpR*+*trpO* insertion |
| 11726 | ACA GTA ATU AAT TAA CCT AGG CTG CTG CCA CC | Forward primer to amplify pCDF-1b together with 11795 for mCherry insertion |
| 11795 | ATG GTA TAU CTC CTT ATT AAA GTT AAA CAA AAT TAT TTC TAC AGG G | Reverse primer to amplify pCDF-1b together with 11726 for mCherry insertion |
| 11830 | ATC GTA CUC TTT AGC GAG TAC AAC CGG G | Reverse primer for amplifying *trpR* together with 11831 to clone with *trpO* on pOSIP-KO-T7 |
| 11831 | AGA GGG CUTTA TCA ATC GCT TTT CAG CAA CAC CTC | Forward primer for amplifying *trpR* together with 11830 to clone with *trpO* on pOSIP-KO-T7 |
| 11832 | AGT ACG AUGAG CTG TTG ACA ATT AAT CAT CGA AC | Forward primer for amplifying *trpO* together with 11833 to clone with *trpR* on pOSIP-KO-T7 |
| 11833 | ATC GTG TTC AUTGT TAT TCT CTA ATT TTG TTC AAA AAA AAG CC | Reverse primer for amplifying *trpO* together with 11832 to clone with *trpR* on pOSIP-KO-T7 |
| 11834 | ATG AAC ACG AUT AAC ATC GCT AAG AAC G | Foward primer to amplify pOSIP-KO-T7 together with 11708 for *trpR*+*trpO* insertion |
| 11910 | AGA CCA GTT UATG AGC AAG GGC GAG GAG GA | Forward primer for amplifying mCherry togther with 11911 to clone on pSEVA27 |
| 11911 | ACC TCA GCUCTA CTT GTA CAG CTC GTC CAT GCC | Reverse primer for amplifying mCherry together with 11910 to clone on pSEVA27 |
| jl17 | ACT GCC UCGAAAGGTTTTGCGCCA | Forward primer to amplify pCDF-mCherry and pCDF-mazF together with jl18 to remove *lacI* |
| jl18 | AGG CAG UGA GCG CAA CGC AAT TAA TG | Reverse primer to amplify pCDF-mCherry and pCDF-mazF together with jl17 to remove *lacI* |
| jl19 | ATA TAC CAUG AGC AAG GGC GAG GAG GA | Forward primer for amplifying mCherry togther with jl35 to clone on pCDF-1b |
| jl26 | ATT TCT TAT CCA UTGT TAT TCT CTA ATT TTG TTC TGT CGA TAC C | Reverse primer to amplify pOSIP-trp-T7 together with pOSIP_UF to remove *trpL* |
| jl35 | AAT TAC TGUCTA CTT GTA CAG CTC GTC CAT GCC | Reverse primer for amplifying mCherry togther with JL19 to clone on pCDF-1b |
| jl106 | ACCTCAGCUCTACCCAATCAGTACGTTAATTTTGGC | Forward primer to amplify *mazF* from the genome together with jl107 to clone on pSEVA27 |
| jl107 | AGACCAGTTUATGGTAAGCCGATACGTACCC | Reverse primer to amplify *mazF* from the genome together with jl106 to clone on pSEVA27 |
| jl108 | AGATATACCAUGGTAAGCCGATACGTACCC | Forward primer to amplify *mazF* from the genome together with jl109 to clone on pCDF-1b |
| jl109 | TAGGTTAATUCTACCCAATCAGTACGTTAATTTTGGC | Reverse primer to amplify *mazF* from the genome together with jl108 to clone on pCDF-1b |
| jl110 | AATTAACCUAGGCTGCTGCC | Forward primer to amplif*y* pCDF-1b together with jl110 for *mazF* insertion |
| jl111 | ATGGTATATCUCCTTATTAAAGTTAAACAAAATTATTTCTAC | Reverse primer to amplify pCDF-1b together with jl111 for mazF insertion |

## Supplementary Figure 1

**Figure S1.** Growth and fluorescence curves in different starting concentrations of tryptophan (trp) or yeast extract (YE). (a) OD and fluorescence for trp-T7 with pSEVA-mCherry. (b) OD and fluorescence for trp*-T7 with pSEVA-mCherry. (c) OD and fluorescence for trp-T7 and pCDF-mCherry. (d) OD and fluorescence for trp*-T7 and pCDF-mCherry. The growth and fluorescence were calculated as the average of three biological replicates.

## Supplementary Figure 2

**Figure S2.** Flow cytometry data from 8 and 24 h of growth in minimal medium. (a) Average fluorescence of pSEVA-mCherry cultures when expressed from the trp-T7, trp*-T7 or lac-T7 expression system. (b) Average fluorescence of pCDF-mCherry cultures when expressed from the trp-T7, trp*-T7 or lac-T7 expression system. (c) Scatterplots showing forward scatter (y-axis) and mCherry fluorescence (x-axis) from pSEVA-mCherry using the trp-T7, trp*-T7 or lac-T7 expression system. (d) Scatterplots showing forward scatter (y-axis) and mCherry fluorescence (x-axis) from pCDF-mCherry using the trp-T7, trp*-T7 or lac-T7 expression system. The trp strains were induced by growth in tryptophan deplete medium, and the lac-T7 system was induced by addition of 1 mM IPTG at OD ~0.3-0.35. OD was similar for all strains at the sampling time points. For (a) and (b), the average values and standard deviations were calculated from three biological replicates. For (c) and (d), each histogram shown is a representative sample out of three biological replicates.
